# Body size influences energetic and osmoregulatory costs in frogs infected with *Batrachochytrium dendrobatidis*

**DOI:** 10.1038/s41598-018-22002-8

**Published:** 2018-02-27

**Authors:** Nicholas C. Wu, Rebecca L. Cramp, Craig E. Franklin

**Affiliations:** School of Biological Sciences, The University of Queensland, Brisbane, Queensland 4072 Australia

## Abstract

Sloughing maintains the skins integrity and critical functionality in amphibians. Given the behavioural, morphological and osmoregulatory changes that accompany sloughing, this process is likely to be physiologically costly. Chytridiomycosis, a cutaneous disease of amphibians caused by the fungus *Batrachochytrium dendrobatidis* (*Bd*), disrupts skin function and increases sloughing rates. Moreover, mortality rates from chytridiomycosis are significantly higher in juveniles and so we hypothesised that smaller individuals maybe more susceptible to chytridiomycosis because of allometric scaling effects on the energetic and osmoregulatory costs of sloughing. We measured *in-vivo* cutaneous ion loss rates and whole animal metabolic rate (MR) of Green tree frogs, *Litoria caerulea*, over a range of body sizes both infected and uninfected frogs during sloughing. Infected animals had a greater rate of ion loss and mass-specific MR during non-sloughing periods but there were no additional effects of sloughing on either of these parameters. There were also significant interactions with body size and *Bd* load indicating that smaller animals with higher *Bd* loads have greater rates of ion loss and higher energetic demands. Our results shed light on why smaller *Bd*-infected anurans often exhibit greater physiological disruption than larger individuals.

## Introduction

Vertebrate skin is a multilayered, semi-permeable tissue providing physical protection from environmental damage, from pathogens and parasites and in some animals, allows the physiological regulation of cutaneous respiratory gases (O_2_, CO_2_), electrolytes (Na^+^, Cl^−^, K^+^) and acid-base (H^+^, HCO^−^) and water balance^1^. In order to maintain the integrity and proper physiological function of the skin, the most superficial layer (*stratum corneum*), is periodically removed and replaced with a new layer through a process known as ‘moulting’, ‘shedding’, or ‘sloughing’^2,3^. Mammals shed skin cells continuously, while birds, snakes and amphibians undergo a cyclic regime of shedding^2^ that may occur as frequently as daily to once or twice a year. For amphibians, sloughing is a physiologically complex process, requiring the integration and synchronisation of circulating hormones^4^ with control of cellular differentiation rates, and the dissolution and reformation of the impermeable skin layers^5^. Skin sloughing has been hypothesised to be energetically costly due to both the physical aspect of removing the slough, and the cellular costs of skin regeneration^6^. In amphibians, sloughing behaviour is largely consistent among species^3,7^ and involves a series of behaviours including hunching, abdominal contractions and limb wiping to remove and eat the old *stratum corneum*^3,8^. Although the energetic cost of sloughing has not been documented in amphibians, it has in snakes. Shedding snakes can increase their metabolic rate (MR) by up to 146% of their standard metabolic rate^9^, and in some species, shedding can be more energetically expensive than digestion and venom production^10^. For endotherms such as penguins and northern elephant seals, a slight increase in energy expenditure over the moulting period does not markedly affect the animals average daily energetic expenditure^11,12^.

While sloughing allows the functionality of the skin to be maintained, there are significant osmoregulatory challenges associated with the removal of the slough for amphibians, such as increased leakage of cutaneous salts and influx of water into the body^13,14^. A substantial component of the energetic cost of sloughing in amphibians likely relates to the complex physiological processes occurring within the new epidermis^5,15^. Specifically, cutaneous osmoregulatory disruptions such as sodium leakage, are compensated for by changing the electrophysiological properties of the skin (reviewed in Erlij)^16^, and increasing the expression of ion transporter proteins post removal of the slough^14^. This leads to an increase in active transcellular ion transport which may also increase the cellular energy expenditure for the tissue^17,18^. Under *in-vitro* conditions, cutaneous active sodium transport can account for ~25% of net O_2_ cutaneous consumption^19^. In addition, the biosynthesis of epidermal tissue also contributes to the cost of sloughing. Although there are no direct studies of the energetic cost of epidermal turnover in amphibians, in sparrows there was a 3.5 fold increase in protein synthesis associated with moulting^20^. The behavioural aspects of sloughing in combination with increased cellular differentiation, compensatory changes in ion transport, and the turnover of the cutaneous surface fluid (mucosal protective layer of the epidermis)^21^ are likely to contribute to the net energetic cost of sloughing and which may represent a considerable component of an amphibian’s overall energy expenditure.

Sloughing may act as an innate immune defence against cutaneous pathogens by increasing the rate of epidermal turnover, and thereby reducing the opportunity for cutaneous pathogens to establish and cause infection^22^. Recently it was shown that in frogs infected with the pathogenic chytrid fungus, *Batrachochytrium dendrobatidis* (*Bd*), sloughing frequency increased significantly^23^. Many anurans infected with *Bd* eventually develop chytridiomycosis, a fatal skin disease responsible for many amphibian declines around the world^24,25^. *Bd* infection also reduces cutaneous sodium uptake capacity and increases passive transcutaneous ion leakage^26^, which, combined with significant inappetence, contributes to the development of low plasma electrolyte levels^27–29^. These behavioural and physiological changes may translate into significant energetic consequences for the infected host. The energetic cost of parasitic infections have been examined in other organisms and may be used a proxy for the impact of infection on overall host physiology including maintenance costs, growth and activity^30^. However, this generalisation about the higher energetic cost of parasitism is not always consistent. In some cases, the MR of parasitised hosts is significantly reduced^31,32^, while in other cases host MR increases^33,34^ or does not change^35^. Such varied responses in MR to parasitism may be due to the context-dependent nature of the energetic costs of parasitism, and may reflect compensatory actions such as changes in host behaviour and physiology that may offset the effects of parasitism on growth and development instead.

*Bd* has been shown to increase the rate of O_2_ consumption (a proxy for MR) in heavily infected frogs^29^ but not in sub-clinically infected frogs^36^. This suggests that *Bd* may not elicit substantial physiological consequences in hosts until infection levels reach a critical threshold^29^. Sloughing, therefore, may be an important immune defence. By increasing the rate of sloughing, the host may constrain *Bd* growth, and thus slow the onset of physiological disruption^7^. The additional effort required to remove the sloughed skin more frequently may increase the overall energy budget of infected frogs and may contribute to the negative health outcomes in susceptible species.

Adverse energetic and osmoregulatory consequences of chytridiomycosis have been observed in a few *Bd* infected anuran species^28,29,37^. These effects may be magnified in smaller frogs, especially juveniles, which often have greater *Bd*-associated mortality rates than larger individuals of the same species^38–41^. Body size (e.g. body mass, snout-vent length, or surface area) dependant mortality rates have been attributed to differences in the maturation of skin immune defences^42^ or immature systemic immune function^39^, differences in skin morphology^43^, diversity of cutaneous microbiomes^44^, and the relative surface area for colonisation. Few studies, however, have directly examined the relationship between body size, *Bd* load and the physiological consequences of *Bd* infection. Metabolic rate and cutaneous surface-area scale allometrically with body mass (exponents of 0.75 and 0.88–0.94, respectively)^45,46^ such that smaller frogs have a greater cutaneous surface area and a higher metabolic rate than larger frogs. Therefore, smaller anurans may be more at risk of cutaneous and metabolic dysfunction relative to larger individuals, and this effect may be exacerbated by increased sloughing rates in infected animals. The present study investigated the effects of body size and *Bd* load on the energetic and osmoregulatory effects of sloughing in the Australian green tree frog (*Litoria caerulea)*. We hypothesised that smaller individuals would experience greater osmoregulatory disturbances and higher mass-specific MR during sloughing than larger individuals. We also predicted that in smaller individuals that develop symptomatic chytridiomycosis, sloughing would exacerbate the osmoregulatory and metabolic effects of *Bd* infection.

## Results

### Intermoult interval (IMI) and number of exposed animals

**Figure 1:**
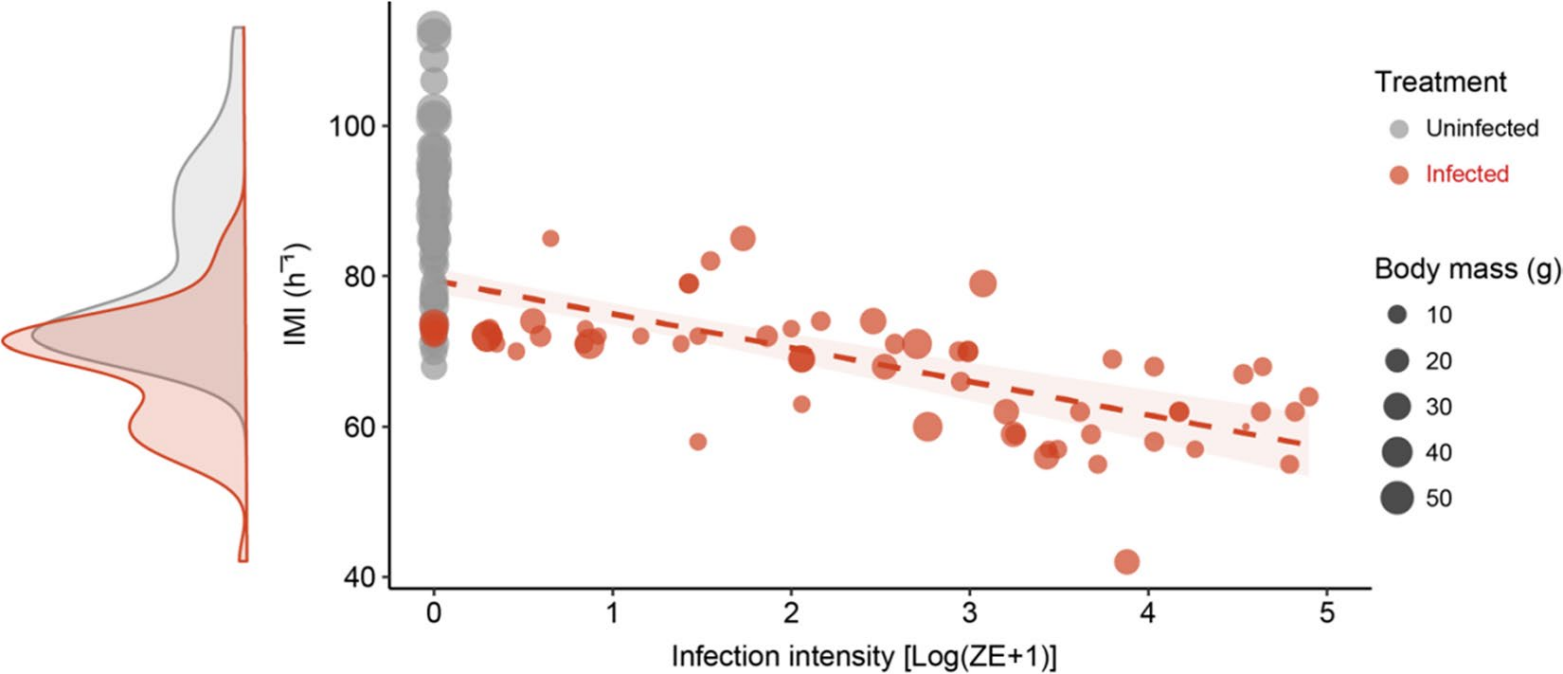
Relationship between intermoult interval (IMI; h^−^¹), body size (g) and *Bd* infection intensity [Log (ZE + 1)] in *Litoria caerulea*. Grey circles () represent uninfected animals and red circles () represent infected animals. Correlation between IMI and *Bd* load is 0.4 with a regression line of *IMI* = −3.26*(ZE* + *1)* + 75.4. Red shading represents 95% confidence interval. The density plot represents distribution of IMI for uninfected and infected animals. Data were presented as individual points of uninfected (*n* = 21) and infected (*n* = 18) animals.

Intermoult interval (IMI) length was positively correlated with body mass (IMI; *t*_146_ = 8, *P* = <0.001; Fig. 1), while *Bd* load was negatively correlated with IMI (Fig. 1; *t*_146_ = −2.3, *P* = 0.02). There was an interactive effect between body mass, IMI and *Bd* load (*t*_146_ = −2.3, *P* = 0.02), with smaller individuals with high *Bd* loads having the shortest IMI (Fig. 1). Animals with high *Bd* loads (above 10,000 ZE) generally stopped performing the characteristic sloughing behaviour, but instead sloughed the *stratum corneum* in small fragments almost continuously.

All animals infected (including re-exposure) either development chytridiomycosis or slowly increased infection load, with the exception of three animals that decreased infection load and recovered in the study.

### Ion loss

**Figure 2:**
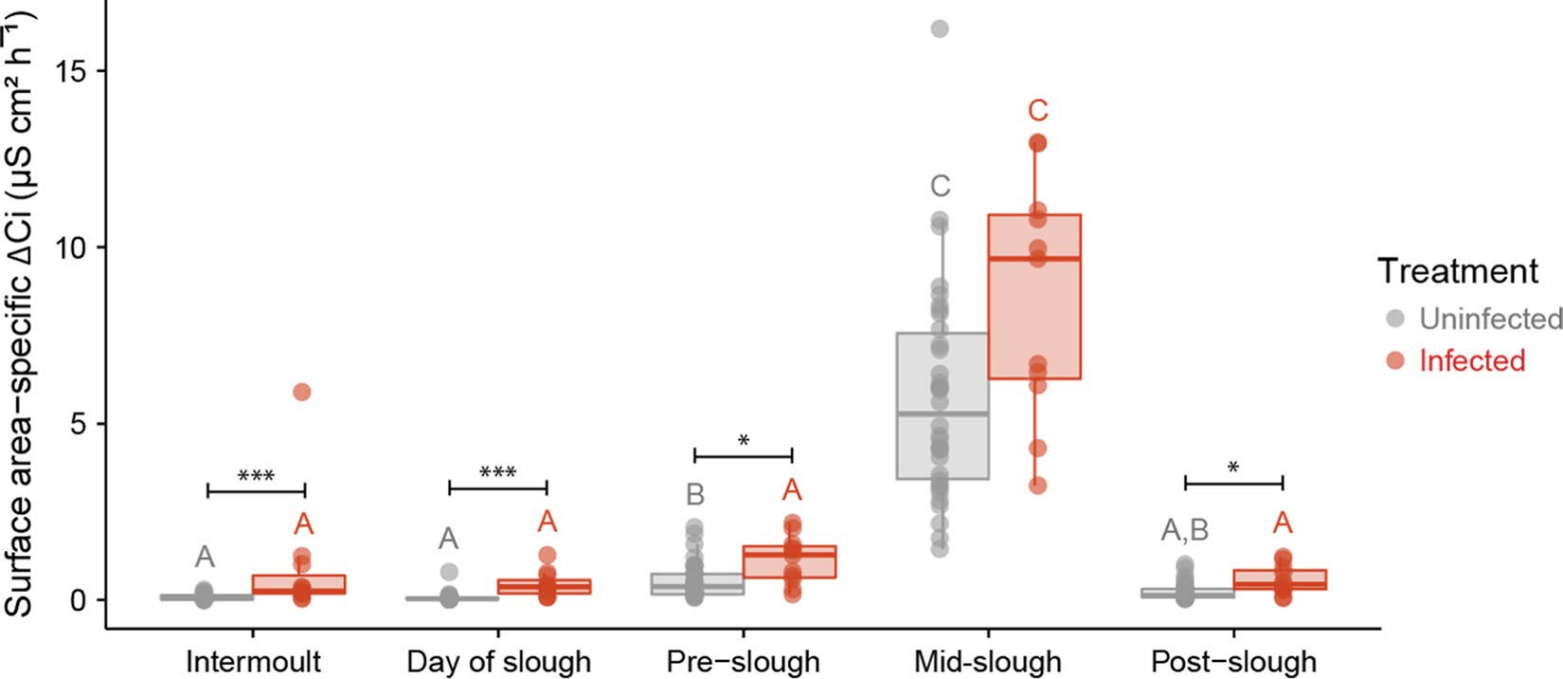
Surface area-specific rate of change in conductivity (Δ Ci; µS cm² h^−^¹) between uninfected and infected *L. caerulea* through the sloughing cycle. Data are presented as individual data points for uninfected (, *n* = 33) and infected (, *n* = 15) animals. Semi-transparent boxplots represent standard distribution of data. Within each treatment (infected or uninfected), slough groups with different letters above them are significantly different from one another. Significant differences between treatments for each slough group were indicated as asterisks. Summary statistics are presented in Tables S1, S2 and S3.

**Figure 3:**
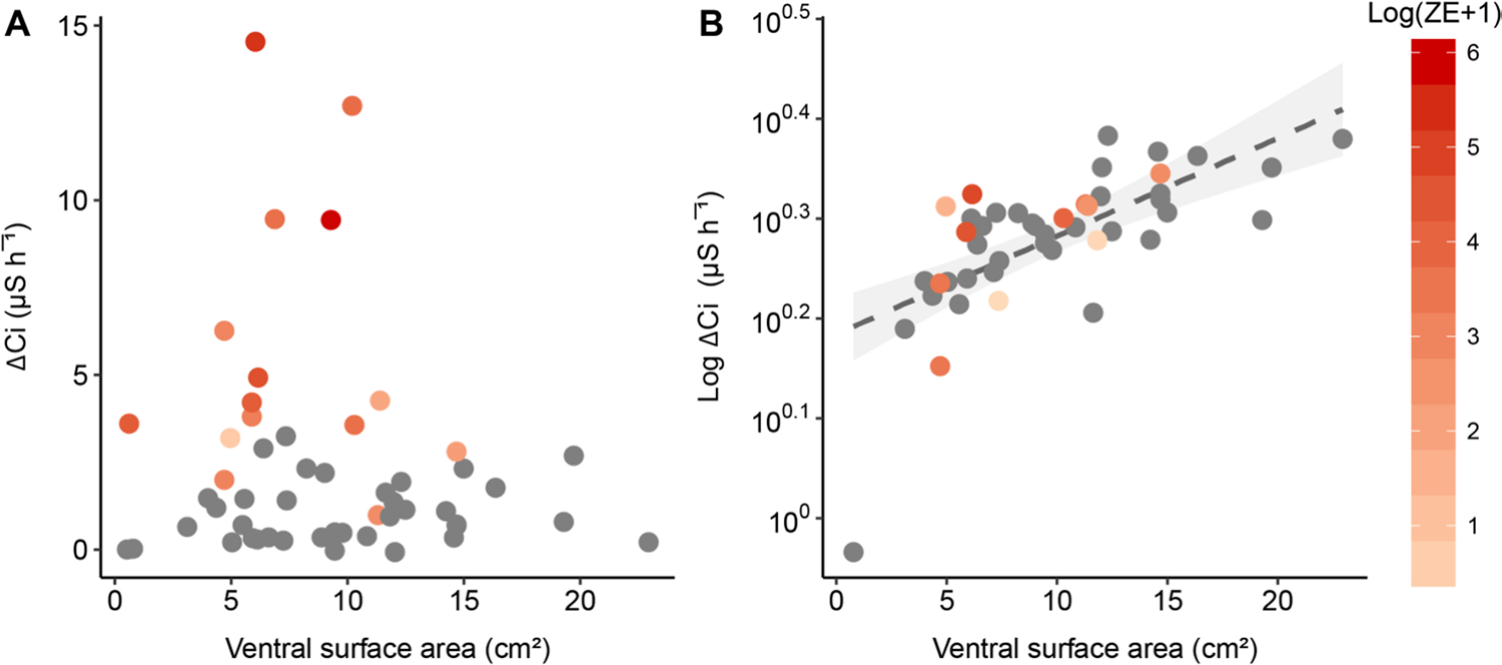
Relationship between rate of change in conductivity (Δ *C*i; µS h^−^¹) and ventral surface area (*A*_V_; cm²) in *L. caerulea* during (**A**) intermoult and (**B**) mid-slough periods. Grey circles () represent uninfected animals and gradient red circles () represent infected animals. Regression line for mid-slough represents an allometric scaling relationship of 0.87 (*C*i = 13*A*_V_^0.87^, R^2^ = 0.72) between surface area and rate of ion loss; grey shading represents 95% confidence interval. Data presented as individual points from *n* = 33 uninfected frogs, *n* = 15 (intermoult) or 10 (mid-slough) *Bd* infected frogs.

The rate of transcutaneous ion loss into the surrounding bath (as evidenced by an increase in conductivity; Δ Ci) was indistinguishable between the intermoult and day of slough groups and post slough groups within uninfected and infected treatments (Tables S1 and S2). Sloughing however, had a significant effect on Δ *C*i increasing by 70 times from 0.14 ± 0.02 µS cm² h^−1^ in intermoult animals to 10.3 ± 0.6 µS cm² h^−1^ during sloughing in uninfected animals (*z* = 10.2, s.e = 0.04, *P* < 0.001; Fig. 2). For uninfected frogs during the intermoult phase, there was no significant correlation between rates of ion loss and ventral surface area (allometric exponent of 0.1; *t*_40_ = 0.4, *P* = 0.7; Fig. 3A). During sloughing, however, ventral surface area had a positive effect on rates of ion loss (*t*_8_ = 6.3, *P* = 0.0002), with an allometric exponent of 0.86 (Fig. 3B).

**Figure 4:**
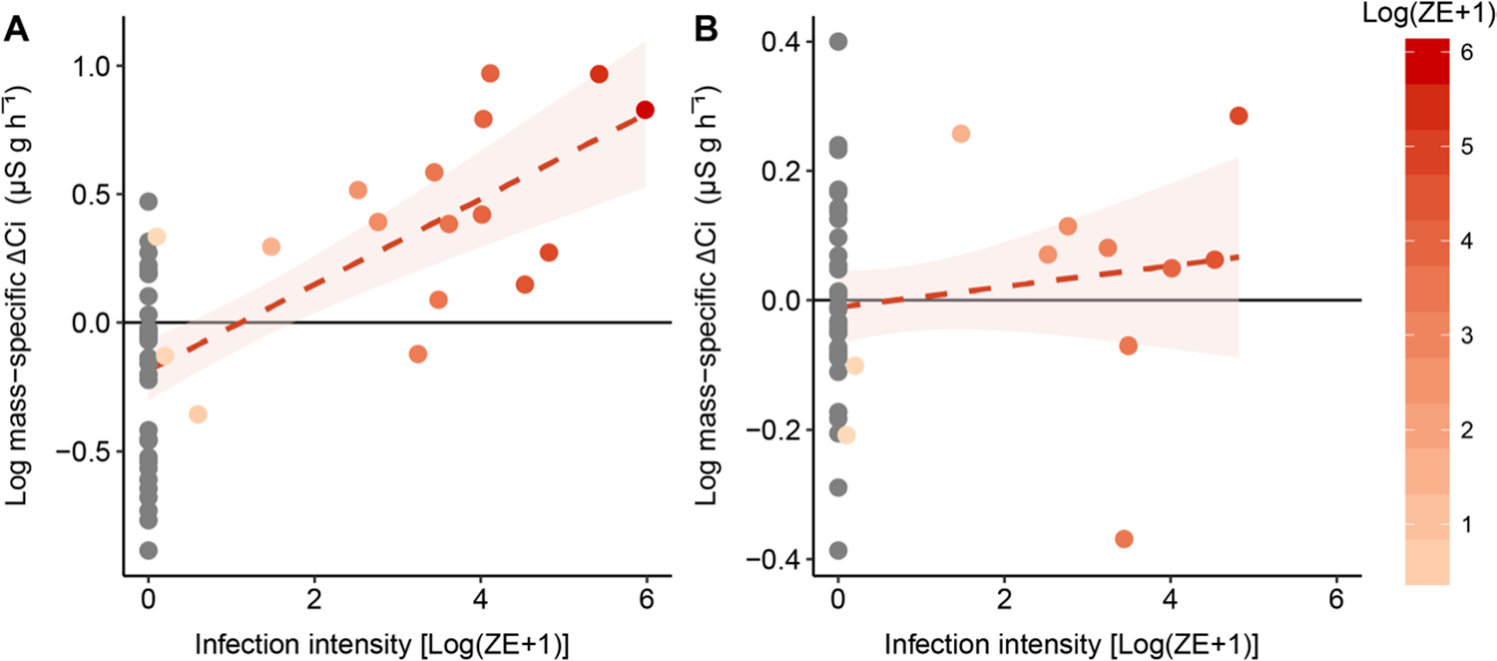
Relationship between mass-specific residuals of rate of change in conductivity (Δ *C*i; µS g h^−^¹) and infection intensity [Log (ZE + 1)] of *L. caerulea* during (**A**) the intermoult and (**B**) mid-slough periods. Grey circles () represent uninfected animals and red gradient circles () represent infected animals. Horizontal line denotes mean residual. Correlation between mass-specific Δ *C*i and infection intensity is 0.38 with a regression line of *Ci* = 0.14 *(ZE* + *1)* − 0.06 for the intermoult group and 0.09 with a regression line of *Ci* = 0.03 *(ZE* + *1)* − 0.08 for mid-slough group. Red area shading around regression line represents 95% confidence interval and data presented as individual points from *n* = 33 uninfected frogs, *n* = 15 (intermoult) or 10 (mid-slough) *Bd* infected frogs.

During the intermoult period, there was a significant effect of *Bd* load on rates of ion loss, with ion efflux rates increasing with increasing *Bd* load (*t*_40_ = 4.1, *P* = 0.002; Fig. 3A). However, rates of ion loss during sloughing in infected and uninfected animals were not significantly different (Fig. 3B). Correcting for body mass, the increase in ion efflux rates with increasing *Bd* load accounted for 39% of the variation during in both intermoult and day of slough stages (Fig. 4A). Like uninfected frogs, frogs infected with *Bd* had a higher rate of ion loss during sloughing (*z* = 8.5, s.e = 0.05, *P* < 0.001), compared to intermoult, day of sloughing and post-sloughing animals (Fig. 2). There was no difference in the rate of ion loss between intermoult, day of sloughing, pre-sloughing and post-sloughing frogs infected with *Bd* (Table S2). When comparing with uninfected animals and accounting for *Bd* infection intensity (*Bd* load in zoospore equivalents, ZE) however, there was a significant interaction between all slough groups (*F*_1,4_ = 4.14, *P* = 0.003), with higher ion loss rates occurring in animals with higher infection loads (Fig. 2; Table S3). Ventral skin surface area did not affect ion loss rates during the non-sloughing phases of the moult cycle of infected animals (*t*_40_ = −0.5, *P* = 0.6), but like uninfected frogs, did significantly affect ion loss rates during the sloughing phase (Fig. 2). However, *Bd* infection did not alter the effect of sloughing on surface area-specific ion loss rates in frogs (Fig. 3B).

Both uninfected and infected animals during sloughing had lower mass-specific ion loss rates compared to the fully permeable agar replica (uninfected *t*_6_ = −13, *P* < 0.001, infected *t*_6_ = −7.8, *P* < 0.001) indicating that ion loss rates were controlled and not unregulated during all phases of the sloughing cycle in both infected and uninfected frogs. The estimated net loss of Na^+^ (mmol h^−1^) over the hour surrounding the sloughing event would represent ~1.2 ± 0.26% (uninfected) and ~0.9 ± 0.4% (infected) of the animals’ total ECF Na^+^ pool. Body size had a significant effect on total Na^+^ loss (*t*_12_ = 4.4, *P* = 0.001), where the rate of Na^+^ loss was greater in smaller animals. The Na^+^ loss between uninfected and infected animals was not significantly different (*t*_12_ = −0.9, *P* = 0.3) and body size did not affect differences in Na^+^ loss between treatments (*t*_12_ = 1.4, *P* = 0.1).

### Energetic cost of sloughing

**Figure 5:**
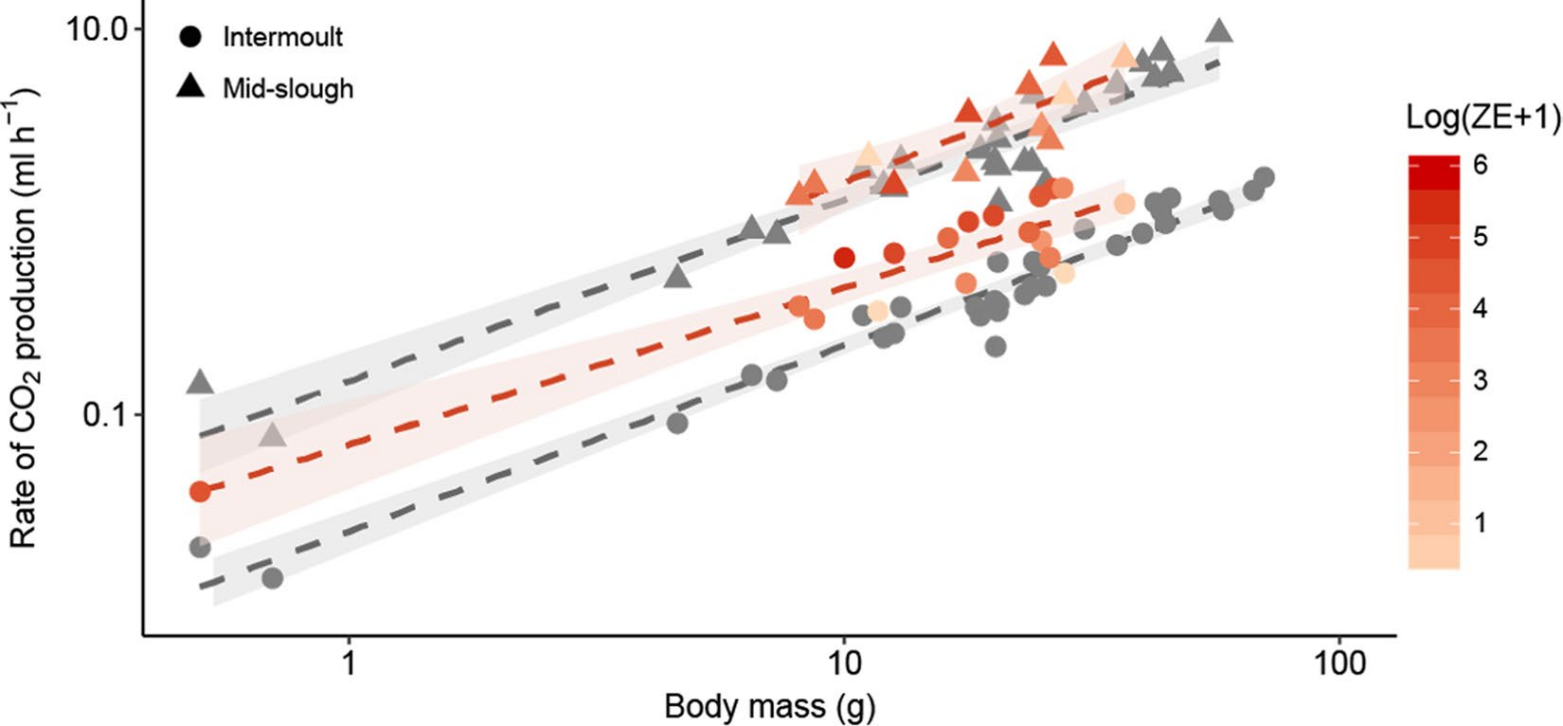
The relationship between rate of CO_2_ production (${\dot{{\boldsymbol{V}}}}_{{\boldsymbol{C}}{{\boldsymbol{O}}}_{2}}$; ml h^−1^) and body mass (*M*_b_; g) of *L. caerulea* during the intermoult and mid-slough periods. Grey circles () represent uninfected animals during intermoult period, grey triangles () represent uninfected animals during mid-slough, red gradient circles () represent infected animals during the intermoult period and red gradient triangles () represent infected animals during mid-slough. Grey dashed regression lines (−) represents the relationship between mass and ${\dot{{\boldsymbol{V}}}}_{{\boldsymbol{C}}{{\boldsymbol{O}}}_{2}}$ of uninfected animals (intermoult ${\dot{{\boldsymbol{V}}}}_{{\boldsymbol{C}}{{\boldsymbol{O}}}_{2}}=0.025{M}_{b}^{0.95}$, R^2^ = 0.95; Mid-slough ${\dot{{\boldsymbol{V}}}}_{{\boldsymbol{C}}{{\boldsymbol{O}}}_{2}}=0.13{M}_{b}^{0.98}$, R^2^ = 0.91), while red dashed regression lines (−) represents the relationship between mass and ${\dot{{\boldsymbol{V}}}}_{{\boldsymbol{C}}{{\boldsymbol{O}}}_{2}}$ of infected animals (intermoult ${\dot{{\boldsymbol{V}}}}_{{\boldsymbol{C}}{{\boldsymbol{O}}}_{2}}=0.06{M}_{b}^{0.8}$, R^2^ = 0.85; Mid-slough ${\dot{{\boldsymbol{V}}}}_{{\boldsymbol{C}}{{\boldsymbol{O}}}_{2}}=0.15{M}_{b}^{1}$, R^2^ = 0.7). Shaded areas around regression lines represents 95% confidence intervals. Data are presented as individual results from *n* = 29 uninfected and *n* = 15 infected intermoult animals and *n* = 28 uninfected and *n* = 10 infected mid-slough animals.

**Figure 6:**
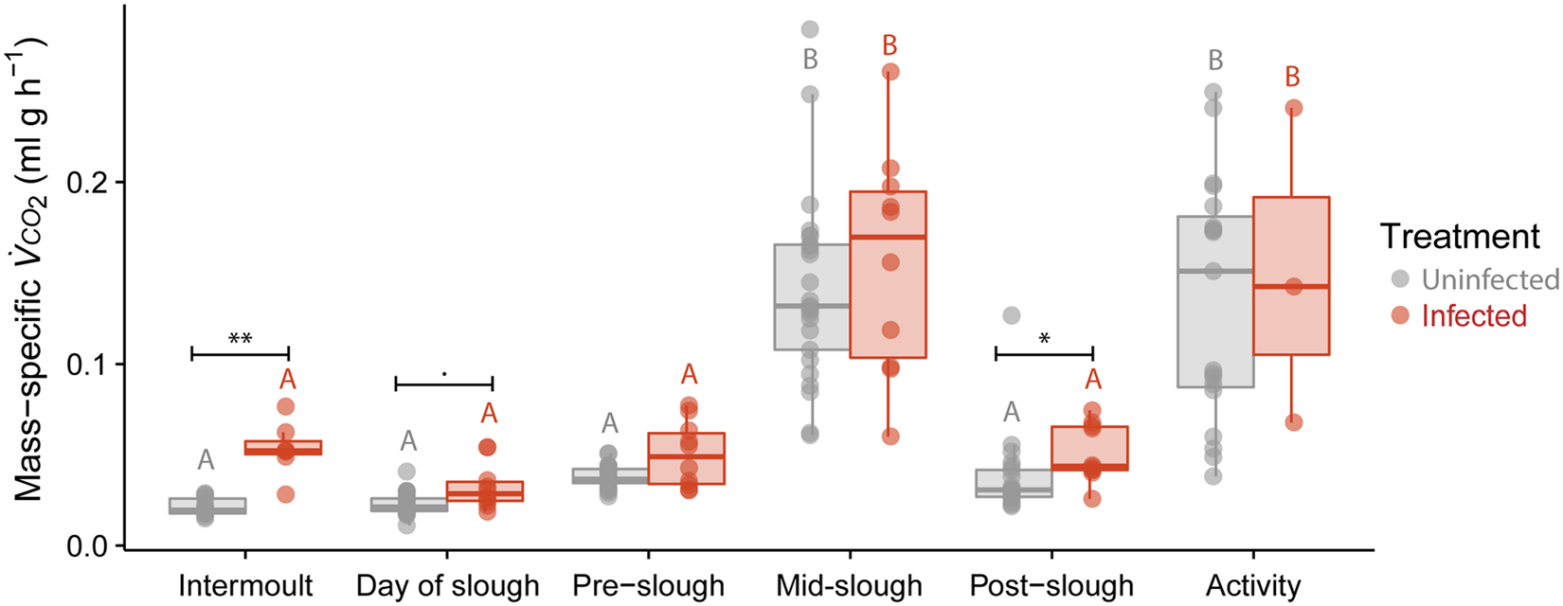
Mass-specific rate of CO_2_ production (${\dot{{\boldsymbol{V}}}}_{{\boldsymbol{C}}{{\boldsymbol{O}}}_{2}}$; ml g h^−1^) between uninfected and infected *L. caerulea* during each recorded behaviour. Data presented as individual data points for uninfected (, *n* = 29) and infected (, *n* = 15) animals. Semi-transparent boxplots represent standard distribution of data. Within each treatment (infected or uninfected), slough groups with different letters above them are significantly different from one another. Significant differences between treatments for each slough group were indicated as asterisks. Summary statistics are presented in Tables S4, S5 and S6.

During the intermoult phase, body mass had a highly significant effect on resting ${\dot{V}}_{C{O}_{2}}$ (*t*_16_ = 10.6, *P* < 0.001; Fig. 5) with an allometric slope of 0.95 (${\dot{V}}_{C{O}_{2}}=0.025{M}_{b}^{0.95}$; R^2^ = 0.95). During sloughing, ${\dot{V}}_{C{O}_{2}}$ was also significantly affected by body mass (*t*_2_ = 13, *P* = 0.005) with a scaling exponent of 0.98 (${\dot{V}}_{C{O}_{2}}=0.13{M}_{b}^{0.98}$; R^2^ = 0.91), as body mass increased, ${\dot{V}}_{C{O}_{2}}\,$increased proportionally. Both uninfected and infected animals during sloughing had significantly higher ${\dot{V}}_{C{O}_{2}}$ than during all other stages of the sloughing cycle (Tables S4 and 5). For uninfected animals, the average rate of CO_2_ production during sloughing was seven times higher than in non-sloughing animals, increasing from 0.02 ± 0.003 ml g h^−1^ during the intermoult period to 0.14 ± 0.01 ml g h^−1^ mid-slough (*z* = 7.2, s.e = 0.006, *P* < 0.001, Fig. 6). This equates to an average energetic cost of 3.0 ± 0.5 J g h^−1^ (Table 1).

**Table 1:** Whole-animal metabolic parameters of infected and uninfected *Litoria caerulea* during the intermoult, mid-slough and active period.

Metabolic parameters							
Treatment	Group	*n*	${\dot{{\bf{V}}}}_{{{\bf{O}}}_{{\bf{2}}}}$	*n*	${\dot{{\bf{V}}}}_{{\bf{C}}{{\bf{O}}}_{{\bf{2}}}}$	RER	MR
			ml g h^−1^		ml g h^−1^	${\dot{{\bf{V}}}}_{{\bf{C}}{{\bf{O}}}_{{\bf{2}}}}$/${\dot{{\bf{V}}}}_{{{\bf{O}}}_{{\bf{2}}}}$	J g h^−1^
Control	Intermoult	8	0.03 ± 0.007	29	0.02 ± 0.001	0.79	0.5 ± 0.02
	Mid-slough	9	0.15 ± 0.03	29	0.14 ± 0.01	1.04	3.07 ± 0.5
	Active	7	0.17 ± 0.02	16	0.13 ± 0.01	0.81	2.8 ± 0.5
Infected	Intermoult	3	0.04 ± 0.01	15	0.03 ± 0.004	1.07	0.70
	Mid-slough	3	0.21 ± 0.05	10	0.16 ± 0.02	0.96	3.58
	Active	0		4	0.14 ± 0.04		

${\dot{{\boldsymbol{V}}}}_{{{\boldsymbol{O}}}_{2}}$= rate of oxygen consumption (ml g h^−1^), ${\dot{{\boldsymbol{V}}}}_{{\boldsymbol{C}}{{\boldsymbol{O}}}_{2}}$= rate of carbon dioxide production (ml g h^−1^), RER = respiratory exchange ratio, MR = equivalent metabolic rate (J g h^−1^), *n* = sample size. Data presented as mean ± s.e.m.

**Figure 7:**
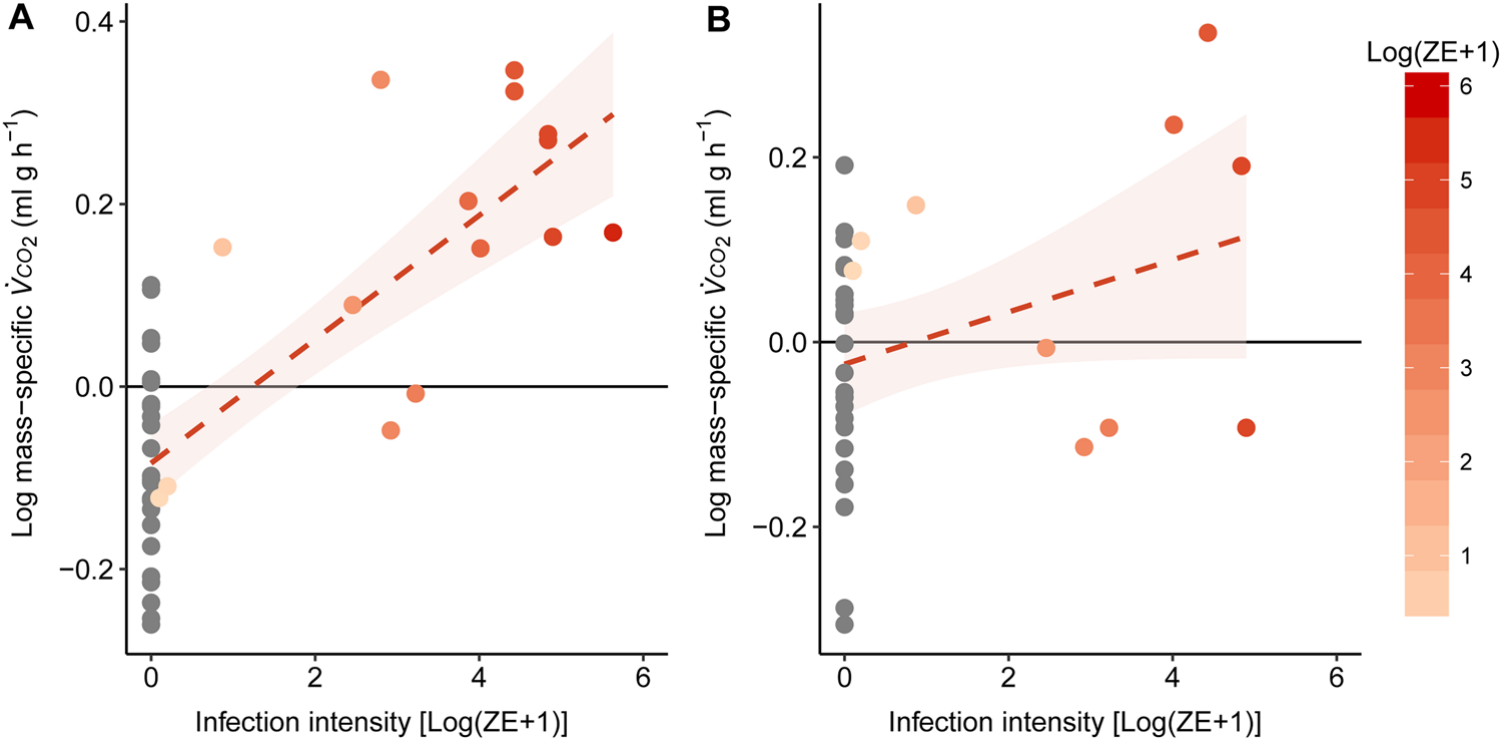
Relationship between mass-specific residuals of rate of carbon dioxide production (${\dot{{\boldsymbol{V}}}}_{{\boldsymbol{C}}{{\boldsymbol{O}}}_{2}}$; ml g h−¹) and infection intensity [Log(ZE + 1)] of *L. caerulea* during (**A**) the intermoult and (**B**) mid-slough periods. Grey circles () represent uninfected animals and red gradient circles () represent infected animals. Horizontal line denotes mean residual. Correlation between mass-specific ${\dot{{\boldsymbol{V}}}}_{{\boldsymbol{C}}{{\boldsymbol{O}}}_{2}}\,$ and infection intensity is 0.46 with a regression line of *Ci* = 0.06*(ZE* + *1)* − 0.05 for the intermoult group and 0.006 with a regression line of *Ci* = 0.006*(ZE* + *1)* − 0.06 for the mid-slough group. Red area around regression line represents 95% confidence interval and data are presented as individual points of intermoult (uninfected *n* = 29, infected *n = *15) and mid-slough (uninfected *n = *28, infected *n* = 10).

While there was a significant main effect of *Bd* load on ${\dot{V}}_{C{O}_{2}}$ (*F*_1,5_ = 17, *P* < 0.001; Table S6), post-hoc analysis revealed that the intermoult and day of slough group showed greatest differences in ${\dot{V}}_{C{O}_{2}}\,$ between infected and uninfected animals (Fig. 6; Table S6). During the intermoult phase, body mass significantly interacted with *Bd* load (allometric slope 0.8; *t*_16_ = 6.2, *P* < 0.001) such that smaller infected animals had higher resting ${\dot{V}}_{C{O}_{2}}$ than larger infected animals (Fig. 5). Moreover, when accounting for body mass, increasing *Bd* load accounted for 65% of the variation in ${\dot{V}}_{C{O}_{2}}$ during the intermoult phase of sloughing (Fig. 7A). However, during sloughing itself, there was no significant effect of *Bd* load on mass-corrected ${\dot{V}}_{C{O}_{2}}\,$ with increasing *Bd* load accounting for ~3% of body-mass corrected ${\dot{V}}_{C{O}_{2}}$ (Fig. 7B). In both infected and non-infected frogs, a typical sloughing event lasting for 5 min would account for <2% of the frog’s total daily (24 h) expenditure (non-infected ~1.38%; infected ~1.46%).

## Discussion

Chytridiomycosis is an epidemic fungal disease affecting around 400 amphibian species worldwide^47^. Understanding its impact on host physiology is important to comprehend both inter- and intra-specific variation in disease susceptibility and morbidity. While sloughing has been shown to contribute to the clearing of *Bd* infections over time in some resistant species, more susceptible species continue to develop chytridiomycosis independent of changes in sloughing frequency^7^. How sloughing and chytridiomycosis may interact in susceptible species to influence the loss of physiological homeostasis in susceptible species, still remains unknown. In the present study, we demonstrated that smaller frogs have shorter IMI than larger individuals, and IMI was further shortened when infected with *Bd*. We also found that frogs infected with *Bd* had a greater rate of cutaneous ion loss and higher resting metabolic rate (RMR) than uninfected individuals. Importantly, this effect was significantly exacerbated by body size, with smaller *L. caerulea* suffering much greater osmotic and metabolic imbalances than larger frogs. Interestingly, the rate of ion loss and metabolic rate (MR) during the actual sloughing event did not differ significantly between infected and uninfected individuals, suggesting that despite significant physical and physiological disruption, *Bd*-infected frogs were able to manage ion efflux to a certain degree during sloughing. However, the degree of physiological disruption from *Bd* increased as animals become more heavily infected, which increases the likelihood that frogs would go onto develop clinical chytridiomycosis.

Body size did not affect rates of net transcutaneous ion loss in uninfected frogs during the intermoult phase, suggesting that healthy amphibians actively regulate cutaneous ion exchange rates to limit excessive ion loss during most of the sloughing cycle^48,49^. By contrast, small *L. caerulea* infected with *Bd* had a greater rate of cutaneous ion loss during the intermoult phase than larger individuals, and the rate of ion loss was exacerbated by increasing *Bd* load. The interaction between body size and *Bd* load may be due to greater surface area (relative to body volume) for *Bd* colonisation in smaller individuals^45^, which is consistent with findings in wild *Mixophyes iteratus* showing that smaller individuals carried higher *Bd* loads than larger frogs^38^. Since *Bd* infects the skin, greater rates of ion loss with higher *Bd* loads might result from direct disruption of skin structure^25,50^ and/or through inhibition of skin ion transport proteins^28^. Similar disruption of other biological processes (reduced body mass, higher RMR and plasma corticosterone and lower lymphocytes) have also be related to *Bd* load^29^, where the greater the *Bd* load, the greater the disruption of homeostatic balance.

The act of sloughing itself increased cutaneous ion-loss rates by 10-fold compared to frogs in the intermoult phase of the slough cycle. Moreover, smaller individuals had disproportionally greater rates of ion loss during sloughing than larger individuals. The relationship between body size and ion loss during sloughing in green tree frogs (exponent of 0.87) is similar to that observed in molting crayfish (*Procambarus clarkii*) (scaling exponent of 0.9)^51^. Although *Bd* load affected rates of ion loss during the non-sloughing phases of the moult cycle, *Bd* infection did not exacerbate ion loss rates, relative to uninfected animals, during sloughing. Under low to moderate infections (<10,000 ZE), animals tend to perform normal sloughing behaviour. However, at high *Bd* loads (>10,000 ZE), animals cease the characteristic sloughing behaviours^23^ and instead the slough detaches almost continuously in fragments^29,50,52^. The increase in ion loss that accompanies sloughing, to some extent, reflects the increase in activity associated with the physical removal of the slough itself. Conceivably then, frogs with heavy *Bd* loads and clinical chytridiomycosis may behaviourally avoid additional ion losses incurred during sloughing by stopping the behavioural process completely. Whether this reflects a deliberate avoidance of ion loss by heavily infected frogs or simply reflects the overall lethargy characteristic of frogs with severe chytridiomycosis^26,52^, remains unclear.

While the skin becomes increasingly ‘leaky’ to ions during sloughing, it does not become fully permeable to ions like in the agar frog replica in this study, suggesting that both infected and uninfected frogs maintain the ability to regulate their rate of ion loss during sloughing. This suggests that even in *Bd* infected frogs, the underlying skin layers maintain a degree of resistance to passive ion loss during sloughing despite the changes that are occurring in the more superficial layers. In addition, sloughing toads increased their cutaneous ion uptake activity and increased skin resistance to control ion losses during sloughing^14^. Relatively lightly *Bd*-infected frogs may also employ compensatory strategies to control ion loss rates during sloughing^28^. This compensation may also come at a physiological cost in the longer term as increasing *Bd* loads stimulate sloughing in *L. caerulea*^23^. Once *Bd* loads reach a threshold intensity, however, control of cutaneous ion loss rates becomes more difficult and animals may stop sloughing altogether to reduce the physiological and energetic costs of sloughing.

Although sloughing only contributed to <2% of their daily expenditure, *Bd*-infected animals slough up to 25% more frequently than uninfected animals^7^. Increased sloughing frequency in *Bd*-infected frogs likely has a cumulative and ultimately detrimental, effect on the physiological balance of frogs infected with *Bd*^29,36^. Increases in both MR and rates of ion loss during sloughing appear to be largely due to the behavioural action of removing the slough. However, the changes in the cellular physiology that accompany sloughing including epidermal cellular proliferation^53^, formation of tight junctions and desmosomes^15^ and upregulating ion and water transport activity to account for leakage of skin^14,54^ also contribute to the increased sloughing-associated energy expenditure.

Non-sloughing green tree frogs infected with *Bd* had a significantly higher RMR compared to uninfected animals, consistent with earlier observations^29^. However, our data show that there was a strong interaction with body size and *Bd* load on RMR, with smaller infected animals having a higher RMR and higher *Bd* loads than large ones. Although RMR is well documented to scale allometrically with body size, our data suggest that not only the ${\dot{V}}_{C{O}_{2}}\,$ intercept increases with *Bd* load, but the slope shifts from 0.96 to 0.8 suggesting smaller infected animals have greater mass-specific metabolic costs. Changes in whole-animal MR in response to a pathogen could indicate the additional costs associated with mounting an immune response^55,56^. *Bd* stimulates a suite of immune responses in infected frogs^57,58^ and the increased immune investment by infected animals may contribute to the increased RMR observed here. Ontological differences in immune responses to *Bd* may also contribute to the differences in mass-specific MR observed^42,58^. The juvenile period is a vulnerable period for infection^42^ and the demand to fight off infection may require that juvenile frogs allocate more energy to the immune responses than older frogs.

Frogs with heavy *Bd* loads have higher rates of ion loss and energy expenditure during non-sloughing periods. Increased rates of ion loss necessitate higher rates of compensatory cutaneous uptake. Under *in-vitro* conditions, the active uptake of 18 Na^+^ ions by healthy amphibian skin costs approximately one mole of O_2_^19,59^, so an increase in cutaneous ion efflux would require a higher cellular respiration rate to maintain salt balance^54^. Since chytridiomycosis also disrupts cutaneous ion uptake capacity^26^, frogs with heavy *Bd* loads may need to invest even more energy in ion uptake to compensate for higher rates of ion efflux and reduced uptake capabilities. In smaller frogs with a large surface area to volume ratio, this effect is likely to be magnified and probably contributes to the higher rates of fatal chytridiomycosis in juvenile frogs.

Our data explain, in part at least, why *Bd*-associated mortality rates are often much higher in juvenile frogs. Smaller (and therefore, younger) anurans carry higher infection loads and are at greater risk of osmoregulatory and metabolic disruptions when infected with *Bd* on account of the allometric scaling of both MR and cutaneous ion loss rates. Although sloughing only transiently increased MR and ion loss rates in frogs, increasing *Bd* load did not exacerbate these changes further. However, the increase in sloughing frequency in small frogs and those with heavy *Bd* infections, may have long-term cumulative effects on the capacity of frogs to maintain homeostatic balance. Further work is needed to determine if the effects observed in the current study are consistent across *Bd* susceptible species and if body size predicts morbidity in *Bd* susceptible species, independent of age.

## Methods

### Ethical statement

All procedures in this study were carried out in accordance with the guidelines and regulations of the approval of The University of Queensland’s Animal Ethics Unit (SBS/316/14/URG) and the Queensland Department of Environment and Heritage Protection (WISP15102214). Full method details are provided in ‘Supplementary Materials’.

### Animal collection and maintenance

*Litoria caerulea* were collected from southeast Queensland and ranged in body mass from 0.5–70 g (*n* = 36). All animals were housed in individual, ventilated, clear plastic containers. Containers were tilted at ~10° with paper towels saturated with chemically aged water (dilution 1:4000; VitaPet, NSW, Australia) as substrate and a half PVC pipe for shelter. The lighting conditions were set at a 12:12 h light-dark photoperiod cycle and temperature maintained at a constant 20.5 ± 0.5 °C. Frogs were checked daily, fed once a week on vitamin-dusted crickets and enclosures cleaned weekly. Prior to experiments, all frogs were swabbed to confirm the absence of *Bd* infection (see below).

### Monitoring sloughing frequency

The intermoult interval (IMI), defined as time between two sloughing events, was monitored continuously using infrared surveillance cameras (model EN-CI20B-65H, Eonboom Electronics Limited; and HW242C Security Camera, K Guard Security, New Taipei City, Taiwan) and a 16 channel H.264 Digital Video Recorder (DVR) system. Each camera monitored two frog enclosures. The videos were extracted daily and the IMI (h) and duration of the sloughing events (min), were calculated following Ohmer *et al*.^7^.

### *Bd* culture and exposure

After one month of monitoring to establish IMI, *Bd* strain EPS4 detailed in Ohmer *et al*.^23^ was used for all experimental infections. A randomised subset of frogs (*n* = 23) were exposed to ~500,000 zoospores. Frogs were exposed for 5 h in 300 ml plastic containers containing 100 ml aged water. Uninfected frogs were treated similarly, but with aged water only. At 2 weeks post-exposure and fortnightly thereafter, each frog was swabbed three times over the frog’s ventral surface, thighs, armpit, forelimb feet, and hindlimb feet^23,38^ to assess infection status. Samples were processed following Boyle *et al*.^60^ and analysed with qPCR in a modified 15 µl reaction^23^. If after one month, exposed frogs had no detectable *Bd* infection, they were re-exposed as above. Infection load or number of zoospore equivalent (ZE) on the skin surface were log + 1 transformed [Log(ZE + 1)]. Prevalence of infection with *Bd* was high: in the first set of infection 69% of exposed frogs developed infection and one month re-exposure 87% of exposed frogs developed an infection. The three frogs that did not develop infection after re-exposure were excluded from the analysis.

### Experimental chronology

Once the IMI was determined for each animal (both uninfected and infected animals), ion loss and respirometry experiments were conducted concurrently (random order) during either the intermoult period or on the predicted day of slough. On the day of experiment, the frogs were swabbed to determine their infection load on that day.

### Ion loss measurements

Changes in ion loss at various points the sloughing cycle were measured as rate of change in the conductivity of a cutaneous freshwater bath (Δ *C*i; microsiemens per hour; Δ µS h^−1^) following Wu *et al*.^14^. Measurements were made and grouped at each of five points in the sloughing cycles: (1) intermoult (half way through slough cycle); (2) The day of sloughing (3 h prior to slough, or on the day of the predicted slough in infected frogs that did not slough); (3) pre-slough (10–20 min prior to slough); (4) mid-slough (during a slough); and (5) post slough (30–60 min post sloughing). Sloughing behaviour was monitored continuously via video surveillance. Each ion loss experiment lasted between 2 and 10 h, depending on when animals sloughed during the timeframe predicted.

Three dimensional (3D) agar models of 4 different sizes of *L. caerulea* were made to examine the effect of surface area on the rate of cutaneous ion loss across a free-flowing permeable surface. Molds of the models were made using Kromopan dental alginate and a 3% agar solution in Ringers solution from Voyles *et al*.^26^ was created in the mold. The agar replicas were placed into water baths and subjected to the same conditions as the living frogs. Ion efflux into the water bath was recorded for 1 h.

The estimated effect of sloughing on an animals sodium budget was calculated as proportion of the total extracellular fluid Na^+^ (as % of ECF Na^+^ h^−1^) following Wu *et al*.^14^ and using plasma samples collected from green tree frogs from a separate experiment. The effective ventral surface area (*A*_v_; cm^2^) indicating body size, across which ion exchanges would occur was calculated by photographing the ventral side of the animal in contact with a glass surface and calculating the surface area (Fig. S1). Images were analysed using Image J (http://imagej.nih.gov/ij/). For surface area-specific ion loss, data was presented as μS cm2 h^−1^.

### Respirometry set-up

Positive pressure flow-through respirometry was used to measure whole animal rates of oxygen consumption (${\dot{V}}_{{O}_{2}}$, ml O_2_ h^−1^) and carbon dioxide production (${\dot{V}}_{C{O}_{2}}$, ml CO_2_ h^−1^), as a proxy for whole animal metabolism (the sum of both respiratory and cutaneous respiration). Dry, CO_2_–free air passed through the metabolic chamber (50 ml, 300 ml or 500 ml glass air-tight container), at a controlled flow rate of either 30 ml min^−1^ (frogs <10 g), 50 ml min^−1^ (10–25 g) or 80 ml min^−1^ (>25 g) and re-scrubbed of water vapour before passing through an infrared CO_2_ gas analyser (LI-820, LI-COR^®^ Biosciences Inc., Lincoln, NE, USA) and an O_2_ analyser (Oxzilla II; Sable Systems, Las Vegas, NE, USA). The fractional concentrations of the CO_2_ and O_2_ in the excurrent air (*F*_*e*_*co*_2_ and *F*_*e*_*o*_2_) were recorded in a PowerLab 4/35 interface and imported into Labchart software (ADinstruments).

### Resting and sloughing metabolic measurements

Each frog was fasted for at least 4 days prior to measurement to ensure a post-absorptive state^61^. Body mass (*M*_b_, g) indicating body size, was recorded before and after the experiment. The resting metabolic rate (RMR) was taken over the period when the animal was in a water conserving posture and behaviourally inactive which corresponded to the lowest O_2_ and CO_2_ readings observed. Metabolic rate during the day of slough, pre-slough, mid-slough and post-slough stages were also recorded. Active MR, defined as the rate of CO_2_ production during a period of continuous movement in the chamber was also measured to compare with the relative energetic cost of sloughing. All activities and behaviours were monitored remotely with a webcam (Microsoft VX-3000) and recorded in Labchart. Respirometry experiments typically lasted between 2–5 h, depending on when animals sloughed during the timeframe predicted. The temperature of the experimental room was maintained at 20.75 ± 0.4 °C. The mean ${\dot{V}}_{{O}_{2}}\,$ and ${\dot{V}}_{C{O}_{2}}$ for all activities were calculated following Lighton^62^. The energy expenditure of mid-slough and activity (J h^−1^) was calculated by subtracting resting ${\dot{V}}_{C{O}_{2}}$ from mid-slough/activity ${\dot{V}}_{C{O}_{2}}$ and multiplying by the energy equivalent of 1 ml CO_2_ production^63^. The estimated cost of sloughing (J g) for an average 5 min sloughing duration relative to their minimal (assuming no activity during the surrounding 24 h period) daily energy expenditure (J g day) was calculated as percentage (%) sloughing expenditure per day.

### Statistical analysis

All analyses were performed in R.3.4.1^64^. Data were presented as means ± standard error (s.e.m.) and α was set at 0.05 for all statistical tests. For the allometric scaling of ion loss and MR, data were log10 transformed and grouped into uninfected and infected. Both ion loss and MR data were first log-transformed to meet the assumptions of normality. All models used a Gaussian error structure.

#### Ion loss

The scaling exponent for ion loss (Ci; Δ µS h^−1^) relative to surface area (*A*_v_; cm^2^) was calculated by linear regression. Differences in Δ *C*i between each slough group were analysed using linear mixed effects (lme) models in the R ‘lme4’ package^65^ with Δ *C*i as the response variable, slough cycle as a fixed effect. *A*_v_ (cm^2^) was used as an additive covariate, and individual identity and number of exposure was included as a random effect to account for repeated measurements on the same individual and the possible effect of exposure. A general linear hypothesis with Tukey contrasts was performed for multiple comparisons of means for both uninfected and infected treatments separately using the R ‘multcomp’ package^66^. To examine interactions between slough groups and *Bd* load [log(ZE + 1)], a linear mixed effects (lmer) model using the R ‘lmerTest’ package^67^ was performed. A summary ANOVA using type 2 Kenward-Roger approximation was performed and post-hoc interactions analysed with Chi squared tests using the R ‘phia’ package^68^. For Δ *C*i in the intermoult and mid-slough group, with the interaction between *Bd* load and *A*_v_, a lme model was performed with *A*_v_ and *Bd* load [log(ZE + 1)] as interactive covariate and individual identity as a random effect to account for repeated measurements within individuals.

#### Metabolic rate

The scaling exponents of ${\dot{V}}_{C{O}_{2}}$ (ml h^−1^) with body mass (*M*_b_; g) were calculated by linear regression. Differences in ${\dot{V}}_{C{O}_{2}}$ between each slough group were analysed using linear mixed effects (lme) models in the R ‘lme4’ package with ${\dot{V}}_{C{O}_{2}}$ as the response variable, slough cycle and active MR as fixed effects. Body mass was included as an additive covariate and individual identity and number of exposure as a random effect to account for repeated measurements on the same individual and the possible effect of exposure. A general linear hypothesis with Tukey contrasts was performed for multiple comparisons of means for both uninfected and infected treatment separately using the R ‘multcomp’ package. To examine interactions between slough groups and *Bd* load [log(ZE + 1)], a linear mixed effects (lmer) model using the R ‘lmerTest’ package^67^ was performed. A summary ANOVA using type 2 Kenward-Roger approximation was performed. Model simplification was then performed using maximum likelihood in ‘lmerTest’ package to eliminate non-significant factors. Due to the non-significant interactive effect of slough groups and *Bd* load in the lmer model (log-likelihood ratio test; χ2 = 1.8, df = 1, *P* = 0.18), it was removed, and the data were refitted to a simplified linear model removing the interactive effects. For ${\dot{V}}_{C{O}_{2}}$ in the intermoult and mid-slough group, and the interaction between *Bd* load and *M*_b_, a lme model was performed with *M*_b_ and *Bd* load [log(ZE + 1)] as interactive covariate, and individual identity as a random effect to account for repeated measurements within individuals.

### Data availability

The datasets generated during and/or analysed during the current study are available from the corresponding author on reasonable request.

## Electronic supplementary material

Supplementary Information
