# Supplementary material for: Body size influences energetic and osmoregulatory costs in frogs infected with Batrachochytrium dendrobatidis
Source: Sci Rep. 2018 Feb 27;8:3739. doi: 10.1038/s41598-018-22002-8 (PMC5829222; doi:10.1038/s41598-018-22002-8)
Supplement: Supplementary file 1 — Supplementary Information [file 41598_2018_22002_MOESM1_ESM.pdf]

Electronic supplementary materials

Title: Body size influences energetic and osmoregulatory costs in frogs infected with *Batrachochytrium dendrobatidis*

Authors: Nicholas C. Wu, Rebecca L. Cramp, and Craig E. Franklin

Methods - Full detail

Animal collection and maintenance

Litoria caerulea spawn was collected from Bribie Island, southeast Queensland in March 2015. Larvae were maintained in aged Brisbane tap water, changed weekly, and fed every second day with frozen spinach. Larvae were reared through to metamorphosis and 19 resulting juveniles (0.5-15 g) were used in subsequent experiments. In addition, 17 juvenile and adult *L. caerulea* (15-70 g) were collected from wet roads in non-protected areas near Fernvale, southeast Queensland in January 2015. All frogs were housed in individual ventilated clear plastic containers, either small (235 x 170 x 120 mm) or large containers (265 x 235 x 12 mm), with paper towels saturated with chemically aged water (dilution 1:4000; VitaPet, NSW, Australia) as substrate, and a half PVC pipe for shelter. Containers were also tilted at ~10° to allow a dry and moist gradient for the frogs to move between. The lighting conditions were set at a 12:12 h light-dark photoperiod cycle, and temperature maintained at a constant 20.5 ± 0.5°C. Frogs were checked daily, fed once a week on vitamin-dusted crickets (*Acheta domesticus*), and enclosures cleaned weekly. Prior to experiments, all frogs were swabbed to confirm the absence of *Bd* infection (see below).

Monitoring sloughing frequency

The intermoult interval (IMI), defined as the period between two sloughing events, was monitored continuously using infrared surveillance cameras (model EN-CI20B-65H, Eonboom Electronics Limited; and HW242C Security Camera, K Guard Security, New Taipei City, Taiwan), and a generic 16 channel H.264 Digital Video Recorder (DVR) system, mounted to a moveable metal frame in front of enclosures, with two cameras per row. Each camera monitored two frog enclosures at one time at a sample rate of 1.56 frames per second. The videos were extracted daily, and the intermoult interval (IMI) (h) and duration of the sloughing event (min), were calculated.

Bd culture and exposure

After one month of monitoring to establish IMI, *Bd* strain EPS4 detailed in Ohmer et al. [1] was used for all experimental infections. Cultures were maintained at 4°C until 4-5 days before exposure. EPS4 isolate was passaged onto sterile 1% agar, 0.5% tryptone, 0.5% tryptone-soy plates and incubated at 20°C. After 4-5 days, zoospores were harvested by flooding plates with aged tap water for 30 min. The zoospore suspension was collected, and the concentration calculated using a haemocytometer [2]. A randomised subset of frogs (n = 23) were exposed to ~500,000 zoospores. Frogs were exposed for 5 h in 300 ml plastic containers containing 100

ml aged water. Uninfected frogs were treated similarly, but with aged water only. At 2 weeks post-exposure and fortnightly thereafter, each frog was swabbed with a sterile fine-tipped cotton swab (MW100-100; Medical Wire & Equipment, Wiltshire, England) three times over the frog's ventral surface, thighs, armpit, forelimb feet, and hindlimb feet [1,3] to assess infection status. Samples were processed following Boyle et al. [2]. Swabs were extracted in 50µl PrepMan Ultra (Applied Biosystems, Foster City, CA, USA), and analysed in duplicate with Taqman qPCR in a thermal cycler (MiniOpticon™ Real-Time PCR Detection System, Bio-Rad Laboratories, Inc.) in a modified 15 µl reaction following Ohmer et al. [1]. If after 1 month, exposed frogs had no detectable *Bd* infection, they were re-exposed as above. Infection load or number of zoospore equivalent (ZE) on the skin surface were log + 1 transformed [Log(ZE+1)]. Prevalence of infection with *Bd* was high: in the first set of infection 65% of exposed frogs developed infection, and one month re-exposure 87% of exposed frogs developed an infection. The three frogs that did not develop infection after re-exposure were excluded from the analysis.

Ion loss measurements

Changes in ion loss during sloughing were measured by placing animals into a ventilated clear chamber (50-1000 ml, depending on the frogs' mass) containing between 30 and 200 ml of distilled water with a magnetic stirrer to circulate the solution. The rate of ion efflux from the animal was measured as change in the conductivity of the bathing solution (microsiemens per hour; Δ µS h^{-1}). Conductivity was measured between two electrodes placed into the solution which were connected to a conductivity pod (ML307, ADInstruments, NSW, Australia). The output was digitised with PowerLab 4/35 interface (ADInstruments) and recorded onto Labchart software (ADInstruments). The baseline of the solution was measured for roughly an hour before the animal was placed into the chamber. Animals were at one of 5 points in their sloughing cycles when measurements were made: 1. intermoult (half way through slough cycle); 2. day of slough (3 h prior to slough, or day of predicted slough in infected frogs but did not slough); 3. pre-slough (10 – 20 min prior to slough); 4. mid-slough (during a slough); and 5. post slough (30 – 60 min post sloughing). Sloughing behaviour was monitored continuously via webcam (Microsoft VX-3000) and measurements for each group was defined: 1. intermoult and 2. day of slough, where the animals remain still; 3. pre-slough, when animals started to extend their limbs to lift the abdomen off the ground; 4. mid-slough which begins with mouth gaping, followed by abdominal contractions, upper body wiping and removal of the old skin; and 5. post-slough, up to 1 h after sloughing when normal behaviour resumes (Fig. S1). All experiments were conducted at room temperature (20.5 ± 0.5°C), and animals were swabbed for *Bd* load prior to the introduction to the chamber.

Three dimensional (3D) agar models of 4 different sizes (20, 40, 60, and 80% of adult size) were made to examine the effect of surface area on the rate of cutaneous ion loss across a free-flowing permeable surface. To make the agar replicas, a life size adult green tree frog plastic model was scanned using a HDI surface scanner on FlexScan3D software (LMI technologies Inc. BC, Canada), then cleaned, refined and filled using Autodesk Meshmixer (Autodesk Inc. CA, USA). The 3D images were then scaled to the appropriate body size by Autodesk 123D Design, and 3D printed via Makerbot Replicator (MakerBot Industries, NY, USA) in Polylactic acid (PLA) filament material. Molds of the models were made using Kromopan dental alginate. A

3 % agar solution in Ringers solution (in mmol l^{-1}: NaCl (112), KCl (2.5), Na_2HPO_4 (2), $CaCl_2$ (1), $MgCl_2$ (1), HEPES salt (5), HEPES (5), pH 7.4, osmolarity 270 ± 20 mOsmol l^{-1}) was poured into the molds and allowed to solidify. The agar replicas were placed into the ion loss water baths and subjected to the same conditions as the living frogs. Ion loss into the water bath was recorded for 1 h.

To estimate the overall effect of sloughing on the animals sodium budget, conductivity measures were converted to a rate of sodium loss (mmol h^{-1}) based on the [Na^+] of bath water samples collected after sloughing (measured via flame photometry (BWB-XP flame photometer; BWB Technologies Ltd, UK)), and assuming that (1) sodium was the primary ion contributing to solution conductivity, and (2) that the proportion of sodium lost relative to other ions was equal [4]. The net amount of Na^+ lost during sloughing was approximated assuming an extracellular fluid (ECF) volume of approximately ~25 % of the body mass of the animal [5]. The actual Na^+ concentration of the ECF was measured from plasma samples collected from green tree frogs in a separate experiment following Wu et al. [4]. The proportion of the total extracellular fluid Na^+ (as % of ECF Na^+ h^{-1}) lost during sloughing was calculated by dividing the rate of ion loss (mmol l h^{-1}) by the total amount of ECF Na^+ (mmol) and multiplied by 100.

The effective ventral surface area (A_v; cm^2) across which ion exchanges would occur was calculated by photographing the ventral side of the animal in contact with a glass surface and calculating the surface area (excluding surface area of limbs and head; Fig. S2). Images were analysed using Image J (http://imagej.nih.gov/ij/). For surface area-specific ion loss, data was presented as μS cm^2 h^{-1}.

Respirometry set-up

Positive pressure flow-through respirometry was used to measure whole animal rates of oxygen consumption ($\dot{V}_{O_2}$, ml O_2 h^{-1}) and carbon dioxide production ($\dot{V}_{CO_2}$, ml CO_2 h^{-1}), as a proxy for whole animal metabolism (the sum of both respiratory and cutaneous respiration). Atmospheric air scrubbed of CO_2 (using soda lime, Chem-Supply, Adelaide, Australia) and water vapour (using Drierite, W. A. Hammond Drierite Co. Ltd, USA) was drawn in to the respirometry system via a sub-sampler pump (SS-3, Sable Systems International, Las Vegas, NV, USA), at a controlled flow rate of either 30 ml min^{-1} (for small frogs < 10 g), 50 ml min^{-1} (for medium frogs 10 – 25 g) or 80 ml min^{-1} (for large frogs > 25 g) by a mass flow controller (GFC17, Aalborg Instruments & Controls Inc., Orangeburg, NY, USA). The dry, CO_2–free air passed through the metabolic chamber (50 ml, 300 ml or 500 ml glass air-tight container (ClipFresh, Hong Kong)), before passing through a relative humidity (RH) analyser (RH-100; Sable Systems, Las Vegas, NE, USA). The air was then re-scrubbed of water vapour before passing through an infrared CO_2 gas analyser (LI-820, LI-COR® Biosciences Inc., Lincoln, NE, USA) and an O_2 analyser (Oxzilla II; Sable Systems, Las Vegas, NE, USA). The fractional concentrations of the CO_2 and O_2 in the excurrent air (F_eco_2 and F_eo_2) were recorded in a PowerLab 4/35 interface and imported into Labchart software (ADinstruments).

The mass flow controller was calibrated using a NIST-traceable bubble film flow meter (1-10-500 mL, Bubble-O-Meter, Dublin, Ohio, USA). The CO_2 analyser was calibrated with dry CO_2-free air and a certified gas mix

(0.386 ± 0.008 % CO_2 in N_2, BOC Gases, Wetherill Park, Australia), and the O_2 analyser was calibrated with dry compressed air (20.5 ± 0.5 % O_2, BOC Gases, Wetherill Park, Australia).

Resting and sloughing metabolic measurements

Each frog was fasted for at least 4 days prior to measurement to ensure a post-absorptive state [6]. The background fractional CO_2 and O_2 concentration of the ex-current air from the respirometry chamber was recorded overnight prior to the introduction of the animal. Body mass (M_b, g) was recorded before and after the experiment, and animals were swabbed prior to the introduction of the chamber. The resting metabolic rate (RMR) was taken over the period when the animal was in a water conserving posture and behaviourally inactive which corresponded to the lowest O_2 and CO_2 readings observed. Metabolic rate during the day of slough, pre-slough, mid-slough, and post-slough stages were also recorded. Active metabolic rate, defined as the rate of CO_2 production during a period of continuous movement in the chamber was also measured to compare with the relative energetic cost of sloughing. All activities and behaviours were monitored remotely with a webcam (Microsoft VX-3000) and recorded in Labchart. The temperature of the experimental room was maintained at 20.75 ± 0.4 °C. The mean $\dot{V}_{O_2}$ and $\dot{V}_{CO_2}$ for all activities were calculated following Lighton [7]:

$$\dot{V}_{O_2} = \mathrm{FR_i}[(F_i\mathrm{O}_2 - F_e\mathrm{O}_2) - F_e\mathrm{O}_2(F_e\mathrm{CO}_2 - F_i\mathrm{CO}_2)]/(1 - F_e\mathrm{O}_2)$$

and

$$\dot{V}_{CO_2} = \mathrm{FR_i}[(F_e\mathrm{CO}_2 - F_i\mathrm{CO}_2) - F_e\mathrm{CO}_2(F_i\mathrm{O}_2 - F_e\mathrm{O}_2)]/(1 - F_e\mathrm{CO}_2)$$

Where $\mathrm{FR_i}$ = incurrent flow rate (ml min), $F_i\mathrm{O}_2$ = incurrent O_2 concentration, $F_e\mathrm{O}_2$ = excurrent O_2 concentration, $F_i\mathrm{CO}_2$ = incurrent CO_2 concentration, and $F_e\mathrm{CO}_2$ = excurrent CO_2 concentration. Instantaneous correction following Seymour et al. [8] was applied to $\dot{V}_{CO_2}$ and $\dot{V}_{O_2}$ to correct for wash-out characteristics:

$$\text{Inst. } \dot{V}_{O_2} \text{or } \dot{V}_{CO_2} = \left[\dot{V}_1 - \dot{V}_2 \times e^{k(t2-t1)}\right]/\left[1 - e^{k(t2-t1)}\right]$$

Where e is the natural logarithm constant (2.71828), k is the washout constant [absolute value of the slope between ln($F_e\mathrm{O}_2$ – $F_i\mathrm{O}_2$) over time], $\dot{V}_1$is the $\dot{V}_{O_2}$or $\dot{V}_{CO_2}$ at time $t1$, $\dot{V}_2$ is the $\dot{V}_{O_2}$or $\dot{V}_{CO_2}$ at time $t2$. The respiratory exchange ratio (RER) was also calculated by dividing $\dot{V}_{CO_2}$ by $\dot{V}_{O_2}$. The energy expenditure of sloughing and activity (J h^{-1}) was calculated by subtracting resting $\dot{V}_{CO_2}$ from sloughing/activity $\dot{V}_{CO_2}$, and multiplying by the energy equivalent of 1 ml CO_2 production (1 ml CO2 = 25.6 J [9]).

Due to inconsistent drifts in the O_2 analyser for some experiments, overall sample size for $\dot{V}_{O_2}$ was low, thus $\dot{V}_{O_2}$data were not used for further statistical analyses. Successful $\dot{V}_{O_2}$were used to calculate RER and converted to energy expenditure. The estimated cost of sloughing (J g) for an average 5 min sloughing duration relative to their minimal (assuming no activity during the surrounding 24 h period) daily energy expenditure (J g day) was calculated as percentage (%) sloughing expenditure per day.

References

1 Ohmer, M. E., Cramp, R. L., White, C. R. & Franklin, C. E. Skin sloughing rate increases with chytrid fungus infection load in a susceptible amphibian. *Funct. Ecol.* **29**, 674-682 (2015).

2 Boyle, D. G., Boyle, D. B., Olsen, V., Morgan, J. A. T. & Hyatt, A. D. Rapid quantitative detection of chytridiomycosis (*Batrachochytrium dendrobatidis*) in amphibian samples using real-time Taqman PCR assay. *Dis. Aquat. Org.* **60**, 141-148 (2004).

3 Kriger, K. M., Hines, H. B., Hyatt, A. D., Boyle, D. G. & Hero, J.-M. Techniques for detecting chytridiomycosis in wild frogs: comparing histology with real-time Taqman PCR. *Dis. Aquat. Org.* **71**, 141 (2006).

4 Wu, N. C., Cramp, R. L. & Franklin, C. E. Fixing a leaky skin: Upregulation of ion transport proteins during sloughing. *J. Exp. Biol.* **220**, 2026-2035 (2017).

5 Hillman, S. S., Withers, P. C., Drewes, R. C. & Hillyard, S. D. *Ecological and environmental physiology of amphibians*. Vol. 1 (Oxford University Press 2009).

6 Secor, S. M., Wooten, J. A. & Cox, C. L. Effects of meal size, meal type, and body temperature on the specific dynamic action of anurans. *J. Comp. Physiol. B* **177**, 165-182 (2007).

7 Lighton, J. R. *Measuring metabolic rates: A manual for scientists*. (Oxford University Press, 2008).

8 Seymour, R. S., Withers, P. C. & Weathers, W. W. Energetics of burrowing, running, and free-living in the Namib Desert golden mole (*Eremitalpa namibensis*). *J. Zool.* **244**, 107-117 (1998).

9 Withers, P. C. *Comparative animal physiology*. (Saunders College Publishing, 1992).

Supplementary figures

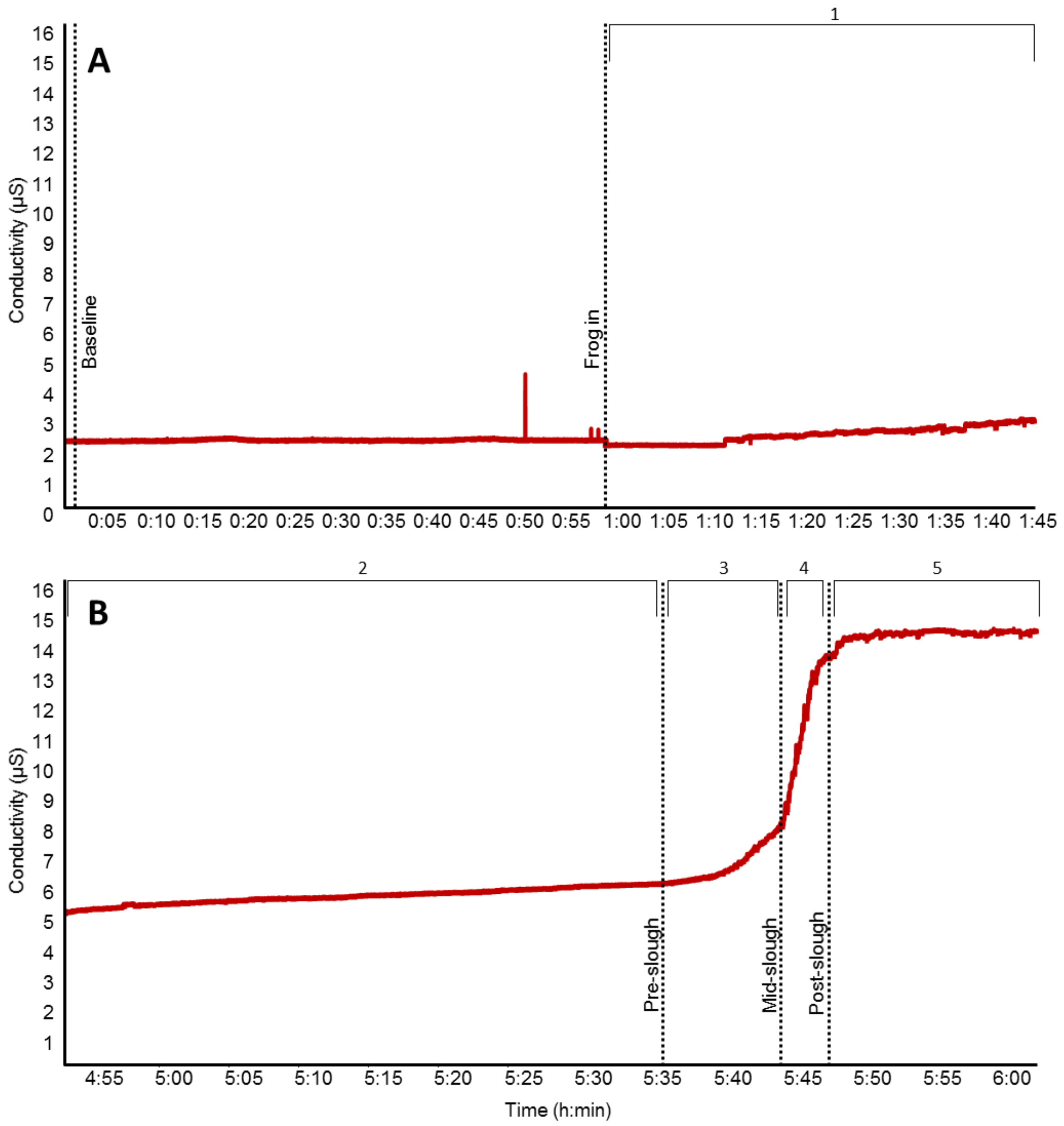

Figure S1. Example raw trace from Labchart of change in conductivity readings for one uninfected *Litoria caerulea* during A) the intermoult period, and B) the sloughing period. Body mass (M_b) = 27.7g, snout-vent length (SVL) = 75.4cm. 'Baseline' values were obtained from the chamber water in absence of animals; 'Frog in' data was when the (1) Intermoult data was obtained with the animal inside the experimental chamber. (2) Indicates 'day of sloughing', (3) indicates 'pre-slough', (4) indicates 'mid-slough', and (5) indicates 'post-slough' period of measurements.

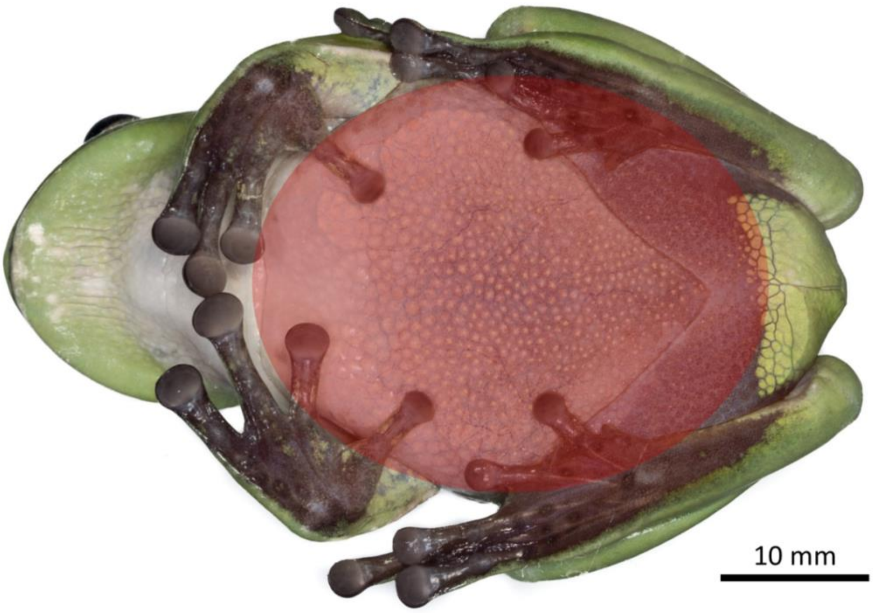

Figure S2. Photograph of a green tree frogs' (*Litoria caerulea*) ventral side in its resting posture. The red semi-transparent circle represents the surface area (cm^2) calculated in Image J.

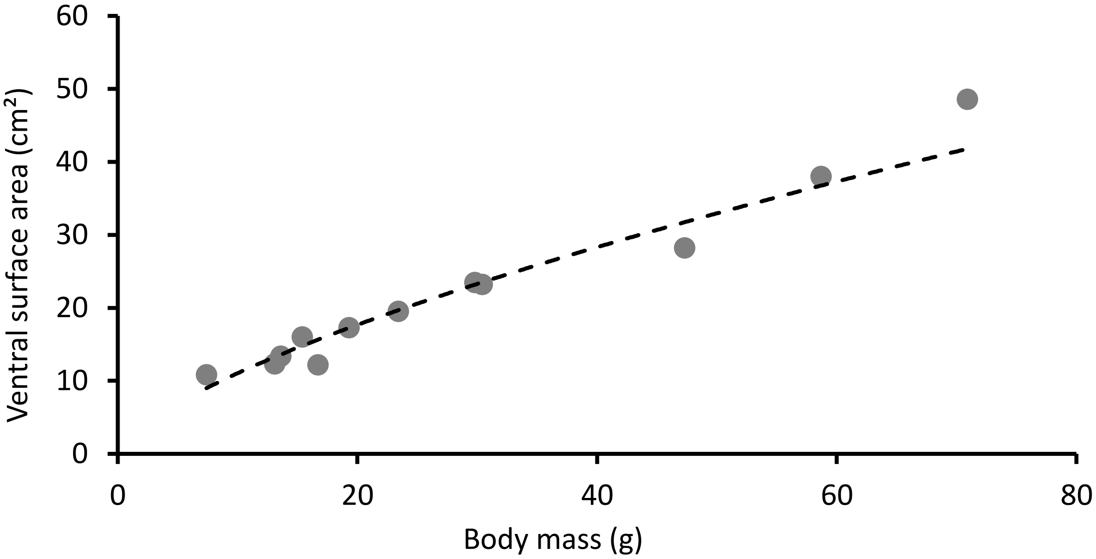

Figure S3. Relationship between ventral surface area (A_v, cm^2) and body mass (M_b, g) of various sized *Litoria caerulea*. Regression line represents an allometric slope of 0.68 (A_v= $2.3M_b^{0.68}$, r^2=0.94).

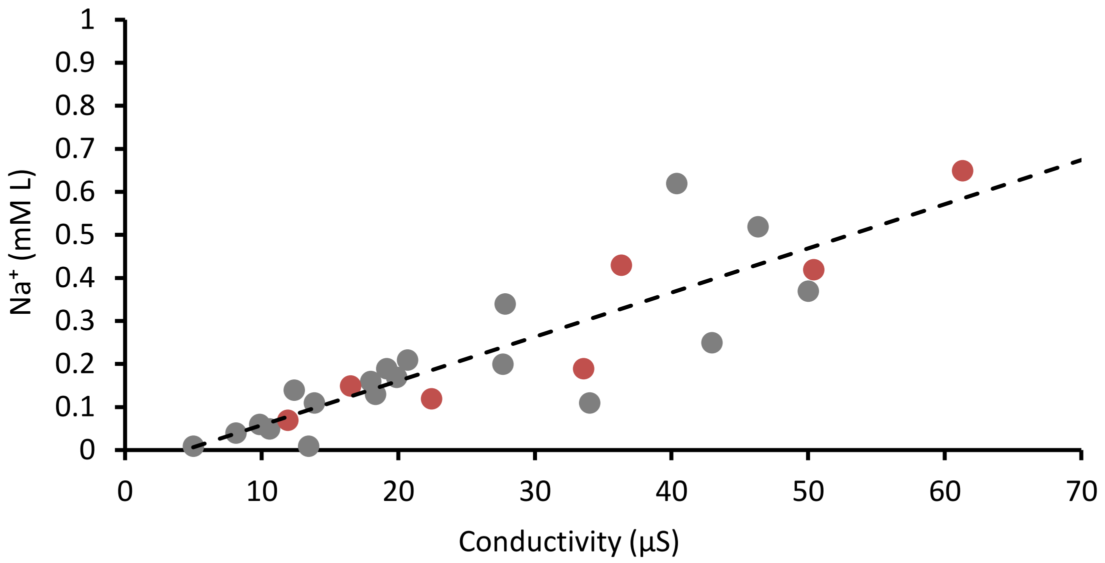

Figure S4. Relationship between sodium (Na^+) levels (mM L) and conductivity (uS) of sloughed water solutions between uninfected (●), and infected (●) *Litoria caerulea*. Correlation between conductivity and Na^+ levels is 0.75 with a regression line of $Na^+ = 0.01(\text{conductivity}) - 0.04$.

Supplementary tables

Table S1. Summary factor interactions from linear mixed effects model for the rate of conductivity between sloughing groups (intermoult, day of slough, pre-slough, mid-slough, and post-slough) for uninfected animals. Covariates were ventral surface area, and frog ID as random variable to correct for repeated measurements**.** *z* value = regression coefficient divided by standard error, s.e.m. = standard error of mean, and Pr(>|z|) = *P* value. Significant codes: 0 '***' 0.001 '**' 0.01 '*' 0.05 '.' 0.1 ' ' 1.

**Groups - Uninfected**					
	**Estimate**	**s.e.m.**	**z value**	**Pr(>|z|)**	
Post-slough - Intermoult	0.05748	0.04789	1.2	0.751	
Pre-slough - Intermoult	0.15283	0.04746	3.22	**0.0113**	*
Day of slough - Intermoult	-0.03306	0.05002	-0.661	0.9645	
Mid-slough - Intermoult	0.48375	0.04746	10.194	**<0.001**	***
Pre-slough - Post-slough	0.09535	0.0451	2.114	0.2136	
Day of slough - Post-slough	-0.09054	0.04741	-1.91	0.3117	
Mid-slough - Post-slough	0.42628	0.0451	9.452	**<0.001**	***
Day of slough - Pre-slough	-0.18589	0.04717	-3.941	**<0.001**	***
Mid-slough - Pre-slough	0.33092	0.04468	7.406	**<0.001**	***
Mid-slough - Day of slough	0.51682	0.04717	10.957	**<0.001**	***

Table S2. Summary factor interactions from linear mixed effects model for the rate of conductivity between sloughing groups (intermoult, day of slough, pre-slough, mid-slough, and post-slough) for infected animals. Covariates were ventral surface area, and frog ID and number of exposure as random variable to correct for repeated measurements and influence of exposure**.** *z* value = regression coefficient divided by standard error, s.e.m. = standard error of mean, and Pr(>|z|) = *P* value. Significant codes: 0 '***' 0.001 '**' 0.01 '*' 0.05 '.' 0.1 ' ' 1.

**Groups - Infected**					
	**Estimate**	**s.e.m.**	**z value**	**Pr(>|z|)**	
Post-slough - Intermoult	0.042014	0.048814	0.861	0.9108	
Pre-slough - Intermoult	0.128043	0.048814	2.623	0.0658	.
Day of slough - Intermoult	0.033768	0.049267	0.685	0.9595	
Mid-slough - Intermoult	0.416905	0.048814	8.541	**<0.001**	***
Pre-slough - Post-slough	0.086029	0.043196	1.992	0.2691	
Day of slough - Post-slough	-0.008425	0.045215	-0.182	0.9998	
Mid-slough - Post-slough	0.374891	0.043196	8.679	**<0.001**	***
Day of slough - Pre-slough	-0.094275	0.045215	-2.085	0.2257	
Mid-slough - Pre-slough	0.288862	0.043196	6.687	**<0.001**	***
Mid-slough - Day of slough	0.383137	0.045215	8.474	**<0.001**	***

Table S3. Summary statistics for rate of conductivity between sloughing groups (intermoult, day of slough, pre-slough, mid-slough, and post-slough) with ZE as interactive effects, and ventral surface area (A_v) as additive effect. Top: Type 2 ANOVA with Kenward-Roger approximation for degrees of freedom between groups, with ZE as interactive effects, and ventral surface area (A_v) as additive effect. Bottom: Post-hoc Chi-square test with Holm-Bonerroni method for the interactions between groups, and treatment accounting for ZE. Sum Sq = sum of squares, Mean Sq = mean squared error, Df = degrees of freedom, NumDF = numerator degrees of freedom, DenDF = denominator degrees of freedom, Chisq = Chi square test, and Pr(>F) & Pr(>Chisq) = *P* value. Significant codes: 0 '***' 0.001 '**' 0.01 '*' 0.05 '.' 0.1 ' ' 1.

Analysis of Variance Table of type II with Kenward-Roger approximation for degrees of freedom							
	**Sum Sq**	**Mean Sq**	**NumDF**	**DenDF**	**F value**	**Pr(>F)**	
Group	338.54	84.635	4	176.8	324.78	**<0.001**	***
Bd	15.09	15.088	1	158.574	57.9	**<0.001**	***
A_v	8.02	8.02	1	38.367	30.78	**<0.001**	***
Group:*Bd*	4.31	1.078	4	181.878	4.14	**0.003126**	**

Chisq Test: P-value adjustment method: holm					
	**Value**	**Df**	**Chisq**	**Pr(>Chisq)**	
Control-Infected : Intermoult	0.246840	1	25.7964	**<0.001**	***
Control-Infected : Day of slough	0.306983	1	39.4300	**<0.001**	***
Control-Infected : Pre-slough	0.159511	1	8.5481	**0.01038**	*
Control-Infected : Mid-slough	0.033617	1	0.3797	0.53778	
Control-Infected : Post-slough	0.140524	1	6.6133	**0.02024**	*

Table S4. Summary factor interactions from linear mixed effects model for the rate of CO_2 production between sloughing groups (intermoult, day of slough, pre-slough, mid-slough, post-slough, and active) for uninfected animals. Covariates were body mass, and frog ID as random variable to correct for repeated measurements**.** *z* value = regression coefficient divided by standard error, s.e.m. = standard error of mean, and Pr(>|z|) = *P* value. Significant codes: 0 '***' 0.001 '**' 0.01 '*' 0.05 '.' 0.1 ' ' 1.

**Groups – Uninfected**					
	**Estimate**	**s.e.m.**	**z value**	**Pr(>|z|)**	
Intermoult – Active	-0.03383	0.0066	-5.126	**<0.001**	***
Post-slough – Active	-0.026	0.005901	-4.407	**<0.001**	***
Pre-slough – Active	-0.02764	0.005647	-4.894	**<0.001**	***
Day of slough – Active	-0.03612	0.005616	-6.431	**<0.001**	***
Mid-slough – Active	0.01168	0.005616	2.08	0.296	
Post-slough - Intermoult	0.007827	0.006403	1.222	0.824	
Pre-slough - Intermoult	0.006189	0.006275	0.986	0.922	
Day of slough - Intermoult	-0.00229	0.006247	-0.366	0.999	
Mid-slough - Intermoult	0.04551	0.006247	7.285	**<0.001**	***
Pre-slough - Post-slough	-0.00164	0.005387	-0.304	1	
Day of slough - Post-slough	-0.01011	0.005354	-1.889	0.406	
Mid-slough - Post-slough	0.037682	0.005354	7.039	**<0.001**	***
Day of slough - Pre-slough	-0.00848	0.005131	-1.652	0.561	
Mid-slough - Pre-slough	0.039321	0.005131	7.664	**<0.001**	***
Mid-slough – Day of slough	0.047797	0.005059	9.448	**<0.001**	***

Table S5. Summary factor interactions from linear mixed effects model for the rate of CO_2 production between sloughing groups (intermoult, day of slough, pre-slough, mid-slough, post-slough, and active) for infected animals. Covariates were body mass, and frog ID and number of exposure as random variable to correct for repeated measurements and influence of exposure**.** *z* value = regression coefficient divided by standard error, s.e.m. = standard error of mean, and Pr(>|z|) = *P* value. Significant codes: 0 '***' 0.001 '**' 0.01 '*' 0.05 '.' 0.1 ' ' 1.

**Groups - Infected**					
	**Estimate**	**s.e.m.**	**z value**	**Pr(>|z|)**	
Intermoult – Active	-0.03567	0.009451	-3.774	**0.00217**	**
Post-slough – Active	-0.03636	0.008595	-4.231	**< 0.001**	***
Pre-slough – Active	-0.03652	0.008601	-4.245	**< 0.001**	***
Day of slough – Active	-0.04557	0.008595	-5.302	**< 0.001**	***
Mid-slough – Active	-0.00361	0.008595	-0.42	0.998261	
Post-slough - Intermoult	-0.0007	0.006881	-0.101	0.999998	
Pre-slough - Intermoult	-0.00085	0.00691	-0.123	0.999996	
Day of slough - Intermoult	-0.0099	0.006881	-1.439	0.694956	
Mid-slough - Intermoult	0.032058	0.006881	4.659	**< 0.001**	***
Pre-slough - Post-slough	-0.00015	0.005659	-0.027	1	
Day of slough - Post-slough	-0.0092	0.005689	-1.618	0.57716	
Mid-slough - Post-slough	0.032756	0.005689	5.758	**< 0.001**	***
Day of slough - Pre-slough	-0.00905	0.005659	-1.6	0.58946	
Mid-slough - Pre-slough	0.032907	0.005659	5.815	**< 0.001**	***
Mid-slough – Day of slough	0.041959	0.005689	7.376	**< 0.001**	***

Table S6. Summary statistics rate of conductivity between sloughing groups (intermoult, day of slough, pre-slough, mid-slough, post-slough, and activity) with ZE as interactive effects, and body mass as additive effect. Top: Type 2 ANOVA with Kenward-Roger approximation for degrees of freedom between groups with ZE as interactive effects, and body mass as additive effect. Bottom: Post-hoc Chi-square test with Holm-Bonerroni method for the interactions between groups, and treatment, accounting for ZE. Sum Sq = sum of squares, Mean Sq = mean squared error, Df = degrees of freedom, NumDF = numerator degrees of freedom, DenDF = denominator degrees of freedom, Chisq = Chi square test, and Pr(>F) & Pr(>Chisq) = P value. Significant codes: 0 '***' 0.001 '**' 0.01 '*' 0.05 '.' 0.1 ' ' 1.

**Analysis of Variance Table of type II with Kenward-Roger approximation for degrees of freedom**							
	**Sum Sq**	**Mean Sq**	**NumDF**	**DenDF**	**F value**	**Pr(>F)**	
Group	23.524	4.7048	5	139.178	90.803	**< 0.001**	***
Bd	0.8827	0.8827	1	96.776	17.036	**< 0.001**	***
Body mass	11.326	11.326	1	40.268	218.593	**< 0.001**	***
Group:*Bd*	0.3212	0.0642	5	147.549	1.24	0.2934	

**Chisq Test: P-value adjustment method: holm**					
	**Value**	**Df**	**Chisq**	**Pr(>Chisq)**	
Control-Infected : Intermoult	0.08487	1	10.1497	**0.008659**	**
Control-Infected : Day of slough	0.06179	1	5.7992	0.064134	.
Control-Infected : Pre-slough	0.03486	1	1.8047	0.420224	
Control-Infected : Mid-slough	0.03012	1	1.3905	0.420224	
Control-Infected : Post-slough	0.07094	1	7.3608	**0.033331**	*
Control-Infected : Activity	- 0.40750	1	2.1771	0.420224	
